# Intravitreal bevacizumab (IVB) versus IVB in combination with pars plana vitrectomy for vitreous hemorrhage secondary to proliferative diabetic retinopathy: a randomized clinical trial

**DOI:** 10.1186/s40942-021-00296-7

**Published:** 2021-04-26

**Authors:** Danilo Moyses Jorge, José Edísio da Silva Tavares Neto, Omero Benedicto Poli-Neto, Ingrid U. Scott, Rodrigo Jorge

**Affiliations:** 1 Department of Ophthalmology, Ribeirão Preto Medical School, University of São Paulo, Av. Bandeirantes 3900, Ribeirão Preto, 14048-900 Brazil; 2 Department of Gynecology and Obstetrics, Ribeirão Preto Medical School, University of São Paulo, São Paulo, Brazil; 3 Departments of Ophthalmology and Public Health Sciences, Penn State College of Medicine, Hershey, PA USA

**Keywords:** Retina, Vitreous, Bevacizumab, Vitrectomy, Vitreous Hemorrhage

## Abstract

**Background:**

The main purpose of this study is to compare the vitreous hemorrhage (VH) score reduction and visual acuity outcomes in patients with VH secondary to proliferative diabetic retinopathy (PDR) treated with intravitreal injections of bevacizumab (IVB) versus IVB and pars plana vitrectomy (IVB and PPV).

**Methods:**

Patients with VH secondary to PDR were randomized into 2 groups: in Group A, patients were treated with a total of 3 IVB (1.5 mg/0.06 ml) at 8-week intervals; and in Group B, patients received a single IVB (1.5 mg/0.06 ml) and, 7 days later, underwent PPV. Patients received an ophthalmic evaluation that included best-corrected visual acuity (BCVA), indirect ophthalmoscopy, and mode B echography at weeks 8, 16 and 24. VH was classified according to the Diabetic Retinopathy Vitrectomy Study classification as grade 1, 2 or 3. Change in VH score was the primary outcome measure and change in BCVA was the secondary outcome.

**Results:**

Seventy-three eyes of 66 patients were randomized and 70 eyes completed the 24-week follow-up visit. Mean VH score reduction (± SEM) of 0.4571 ± 0.0283 (*p* = 0.0014), 1.3429 ± 0.0393 (*p* < 0.0001) and 1.8286 ± 0.0438 (*p* < 0.001) was observed in Group A at 8, 16 and 24 weeks after treatment, respectively (Table 2; Fig. 2). In Group B, the reduction of VH score (± SEM) was 2.2571 ± 0.0720 (*p* = 0.0014), 2.2857 ± 0.0606 (*p* < 0.0001) and 2.2286 ± 0.0726 (*p* < 0.001) at 8, 16 and 24 weeks after treatment, respectively. Group comparison revealed a significantly greater reduction in mean VH score in Group B at 8 and 16 weeks after treatment (*p* < 0.0001). However, at 24 weeks this difference was no longer statistically significant (*p* = 0.1854). In Group A, mean (± SEM) BCVA showed an improvement of 0.00285 ± 0.0004 (*p* = 0.971), 0.5371 ± 0.0072 (*p* < 0.0001), 0.8143 ± 0.0001 (*p* < 0.0001) and 0.8543 ± 0.0008 (*p* < 0.0001) compared to baseline at 1, 8, 16 and 24 weeks after treatment, respectively. In Group B, mean (± SEM) BCVA showed an improvement of 0.3657 ± 0.0507 (*p* = 0.0002), 0.8857 ± 0.0385 (*p* < 0.0001), 0.9457 ± 0.0499 (*p* < 0.0001) and 0.9629 ± 0477 (*p* < 0.0001) compared to baseline at 1, 8, 16 and 24 weeks after treatment, respectively. No significant difference in BCVA improvement was observed between groups at 24 weeks after treatment.

**Conclusion:**

PPV with preoperative IVB is associated with more rapid clearance of VH and improvement in BCVA than IVB injections alone. However, after 24 weeks of follow-up, the reduction in VH score and BCVA were similar between both treatment strategies.

Trial Registration

The project is registered in Plataforma Brasil with CAAE number 927354.7.0000.5440 and was approved by the Ethics Committee of the Clinics Hospital of Ribeirao Preto Medicine School of São Paulo University—Ribeirão Preto, São Paulo, Brazil (appreciation number 3.053.397 gave the approval).

## Background

Diabetic retinopathy (DR) is the leading cause of blindness among working aged individuals in developed countries. After 15 years of diabetes mellitus (DM), 80% of patients with type 2 DM and 97% of those with type 1 DM are estimated to have some degree of retinopathy [1] and, without treatment, 50% of individuals with the proliferative form of the disease are expected to be blind within 5 years [2].

The physiopathology starts with the persistent hyperglycemia that commonly affects diabetic patients, inducing retinal hypoxia and triggering the production of vasoactive factors that may lead to macular edema and/or angiogenesis, with the presence of retinal neovascularization (NV) representing an important risk factor for loss of vision in patients with DR [3, 4].

Proliferative diabetic retinopathy (PDR) is an important cause of severe visual loss in patients with DM [3]. Panretinal laser photocoagulation (PRP) is the standard treatment for retinal and optic disc neovascularization; approximately 60% of patients respond to PRP with regression of NV within 3 months [5]. However, in some cases, complete NV regression does not occur after PRP and 4.5% of patients require pars plana vitrectomy (PPV) despite PRP.

Vascular endothelial growth factor (VEGF) plays an important role in the microvascular complications of the retina [6–8], with its levels being approximately three times higher in patients with PDR compared to nondiabetic individuals and triggering the development of NV [9, 10]. High VEGF levels inducing NV is also observed in other retinal vasculopathies that lead to ischemia, such as central retinal vein occlusion. Abnormal and incompetent blood vessels may grow along the posterior surface of the vitreous, causing bleeding inside the vitreous cavity (vitreous hemorrhage) and/or fibrovascular proliferation culminating in traction retinal detachment [11].

Vitreous hemorrhage (VH) may be traumatic or spontaneous, with PDR accounting for 32% of spontaneous cases [12]. The standard examination for VH evaluation is A and B mode ocular echography [13]. According to the 1985 Diabetic Retinopathy Vitrectomy Study (DRVS) [14], VH does not resorb spontaneously in 80% of patients and requires surgery, with pars plana vitrectomy (PPV) being the procedure of choice. However, re-bleeding is observed in 20–40% of patients even after surgery [11].

Regression of optic disc NV was demonstrated after intravitreal injection of the antiangiogenic agent bevacizumab (Avastin®; Genentech, Inc.; South San Francisco, CA, USA) within the context of DR [15, 16]. However, this effect seems to be transient since NV tended to recur 12 weeks after a single intravitreal injection of bevacizumab. [17].

The administration of anti-VEGF agents such as bevacizumab and ranibizumab has shown promising results in the management of dense VH, shortening the time for VH clearance and reducing the need for PPV by about 30% [18, 19].

For patients with PDR who undergo PPV, several studies have shown the importance of injecting an anti-VEGF agent within up to 7 days before PPV in order to avoid intra and postoperative bleeding [20, 21].

The purpose of the current study was to compare the VH score reduction and rate of recurrent VH in patients with VH secondary to PDR treated with intravitreal injections of bevacizumab (IVB) versus IVB and PPV.

## Methods

After approval from the Local Human Research Ethics Committee was obtained, the study was conducted between January 1, 2019 and December 31, 2019 according to the Declaration of Helsinki.

### Inclusion and exclusion criteria

Inclusion criteria were: patients older than 18 years, VH duration of more than 3 months, and visual acuity worse than 20/40 in the study eye. These characteristics were confirmed by indirect ophthalmoscopy and/or ocular echography. All selected subjects gave written informed consent to participate in the study.

Exclusion criteria were: intraocular surgery during the last 3 months, previous PPV, acute ocular infection, associated traction retinal detachment, clinically uncontrolled glaucoma, severe recent ocular trauma, use of anticoagulant medications (except aspirin), glycosylated hemoglobin of more than 13%, any condition that would affect documentation or follow-up, and participation in another clinical study within the last 30 days.

During the recruitment phase, 73 eyes of 66 patients who met the study criteria were enrolled into the study. The ophthalmological examination conducted during the initial evaluation of both groups consisted of best-corrected visual acuity (BCVA) recorded with a logMAR table according to the standardized recommendations of the Early Treatment Diabetic Retinopathy Study (ETDRS), with the exception that hand movements and counting fingers were also employed as visual acuity measurements, when the patient could read chart letters at 1 m. Applanation tonometry with a Goldmann tonometer, binocular indirect ophthalmoscopy, color fundus photography, and mode B echography were performed in the study eye. On subsequent follow-up examinations, patients underwent the same ophthalmological examination except for ocular B echography. The examiner who measured BCVA and graded the VH score at visits 8, 16 and 24 was masked and was unaware of the group to which each patient belonged.

### Randomization and treatment group

Patients were randomized into the following two groups: Group A, in which the patients received a total of 3 intravitreal injections of 0.06 ml (1.5 mg) bevacizumab (Avastin®) administered at 8-week intervals; and Group B in which the patients were treated with a single injection of 0.06 ml bevacizumab (1.5 mg) 7 days before undergoing PPV. Patients in Group A received PRP as soon as clearing of the VH permitted; in Group B, endolaser panphotocoagulation was performed during PPV.

### Intravitreal injection

Bevacizumab (1.5 mg in 0.06 ml) was administered via a disposable BD Ultra-Fine™ 29G ½ syringe through the pars plana, under topical anesthesia, 3 mm from the limbus in pseudophakic patients and 3.5 mm from the limbus in phakic patients. After the procedure, perfusion of the optic nerve was confirmed by indirect ophthalmoscopy. Patients in Group A were instructed to use antibiotic eyedrops (0.5% moxifloxacin), one drop every 6 h in the study eye, starting three days before the injection for prophylaxis and continuing for one week after injection. Groups B patients were instructed to follow the standard post-PPV eyedrop regimen: moxifloxacin 1 drop qid for 1 week and dexamethasone 1 drop q6h for 1 week with progressive reduction in drop frequency for 1 month.

### Standardization of vitreous hemorrhage grades and other outcomes measures

VH was classified according to the DRVS: grade 1 when details of the retina were visualized with the aid of a binocular indirect ophthalmoscope (BIO), grade 2 in the presence of a red reflex but with retinal details impossible to visualize, and grade 3 in the presence of VH with the absence of a red reflex upon examination with a BIO. Patients with grade 3 VH were examined with ocular ultrasound to look for retinal detachment. Change in VH score was the primary outcome measure and change in BCVA was a secondary outcome measure.

### Pars plana vitrectomy

As per usual preoperative protocol at our institution, medical examination and assessment of surgical cardiology risk were obtained for all patients. The type of anesthesia used was left to the discretion of the anesthesia service according to the standards normally used for PPV, with peribulbar blockade with 6 ml 1% ropivacaine being usually employed. The surgical technique consisted of phacoemulsification with intraocular lens implantation (if the patient was phakic) and 23-gauge PPV, partial fluid-air exchange and endolaser PRP until the *ora serrata* if the patient had not yet received complete PRP. Perfluropropane (C3F8) or silicone oil could be used in cases of retinal tears at the surgeon’s discretion.

### Ophthalmologic evaluation

Patients in Group A underwent ophthalmologic examination including ocular echography in the eye with VH, and were then treated with intravitreal injection of 0.06 ml (1.50 mg) bevacizumab. The patients were followed up with serial ophthalmologic examinations at 1 day and at 1, 3, 8, 16 and 24 weeks after the procedure, with additional bevacizumab injections administered at 8 and 16 weeks, for a total of 3 injections.

Patients in Group B underwent ophthalmological examination including ocular echography in the eye with VH, and were then scheduled for PPV. The patients were followed up with serial ophthalmologic examinations at 1 day and at 1, 3, 8, 16 and 24 weeks after surgery.

### Statistical analysis

We used SAS (version 9.3) for statistical analysis. Quantitative variables with skewed distribution were analyzed using the Mann–Whitney test. A mixed effects linear regression model was applied to determine the effect of time on the outcomes. Each time point and each group were compared by orthogonal contrast analysis.

### Sample size estimation

A computerized search of the Medline database revealed no studies that presented statistics with means and standard deviations or medians and ranges or confidence intervals for VH scores. For this reason, an exploratory analysis was conducted with a small sample size to test this parameter.

## Results

**Fig. 1:**
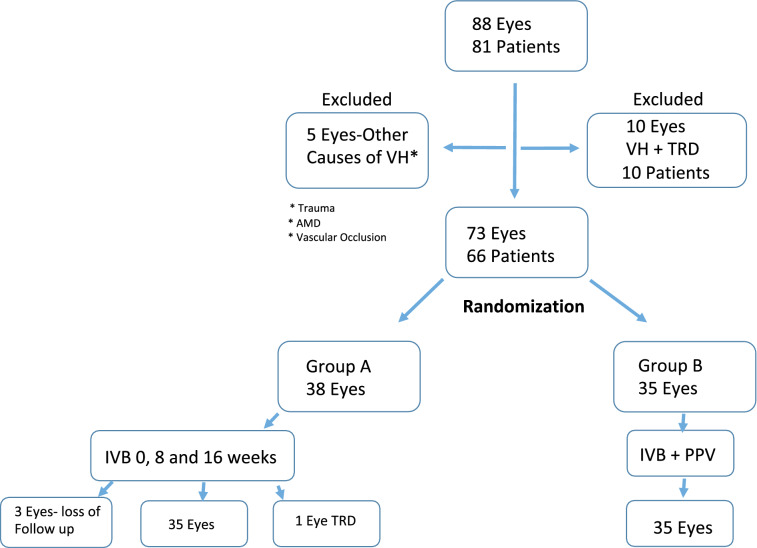
Patients’ flow diagram

Eighty-eight eyes in 81 patients presented to our retina service with visually significant VH during the study period. Five were excluded because the VH was not related to PDR. Among the other causes of VH were wet age-related macular degeneration (AMD), trauma and vascular occlusions. Ten eyes/patients were excluded after ocular echography demonstrated tractional retinal detachment (TRD) associated with the VH. Seventy-three eyes of 66 patients older than 18 years with VH secondary to PDR were randomized to the two study groups. Group A included 38 eyes and Group B included 35 eyes. Three patients from Group A missed 2 consecutive study visits and were excluded. One patient from Group A developed TRD after his first anti-VEGF injection and underwent PPV, endolaser PRP and silicone oil injection. Despite this complication, his data were included in the final analysis. At visit 24, 35 eyes from each group were included in the analysis. (Fig. 1) Seven patients have both eyes included in the study, one in each group. The demographic characteristics of the patients are summarized in Table 1, with no significant difference in age, gender or presence of systemic arterial hypertension between groups. Table 1 also displays the VH score at baseline, with no significant difference between groups (*p* > 0.05). Median logMAR BCVA at baseline was 1.9 (20/1600 or CF 2 m), range: 2.1 [HM] to 0.5 [20/63] and 2.0 (20/2000 or CF 1 m) (range: HM [2.1] to 0.6 [20/80] in Groups A and B, respectively (*p* = 0.013).

**Table 1:** Patient’s demographic characteristics and vitreous hemorrhage score at baseline

	Group A	Group B	*p*
Age (Mean ± SD)	63.66 ± 8.16	64.03 ± 11.24	0.8475
Gender	15 M//20 F	18 M//17 F	0.1470
SAH (n)	34	34	0.5072
Lens Status (phakic / pseudophakic)	28 (80.0)/7 (20.0)	31 (88.58)/4 (11.42)	
Previous PRP (%)	16 (45.7)	13 (37.1)	0.4667
Partial PRP *	8 (22.85)	9 (25.8)	
Complete PRP	8 (22.85)	5 (14.3)	
Baseline VH Score (%)			0.3735
Grade 1	3 (8.6)	1 (2.9)	
Grade 2	14 (40)	11 (31.4)	
Grade 3	18 (51.4)	23 (65.7)	

*SAH* systemic arterial hypertension, *M* male, *F* female, *PRP* panretinal photocoagulation, *VH* vitreous hemorrhage

* ≤ 3 quadrants of full scatter PRP

### Vitreous hemorrhage scores

The distribution of the VH scores for Groups A and B is presented in Tables 1, 2, and 3. At baseline, the median score of VH was 3 (mean: 2.416) and 3 (mean: 2.578) for Group A and B, respectively (*p* < 0.001).

**Fig. 2:**
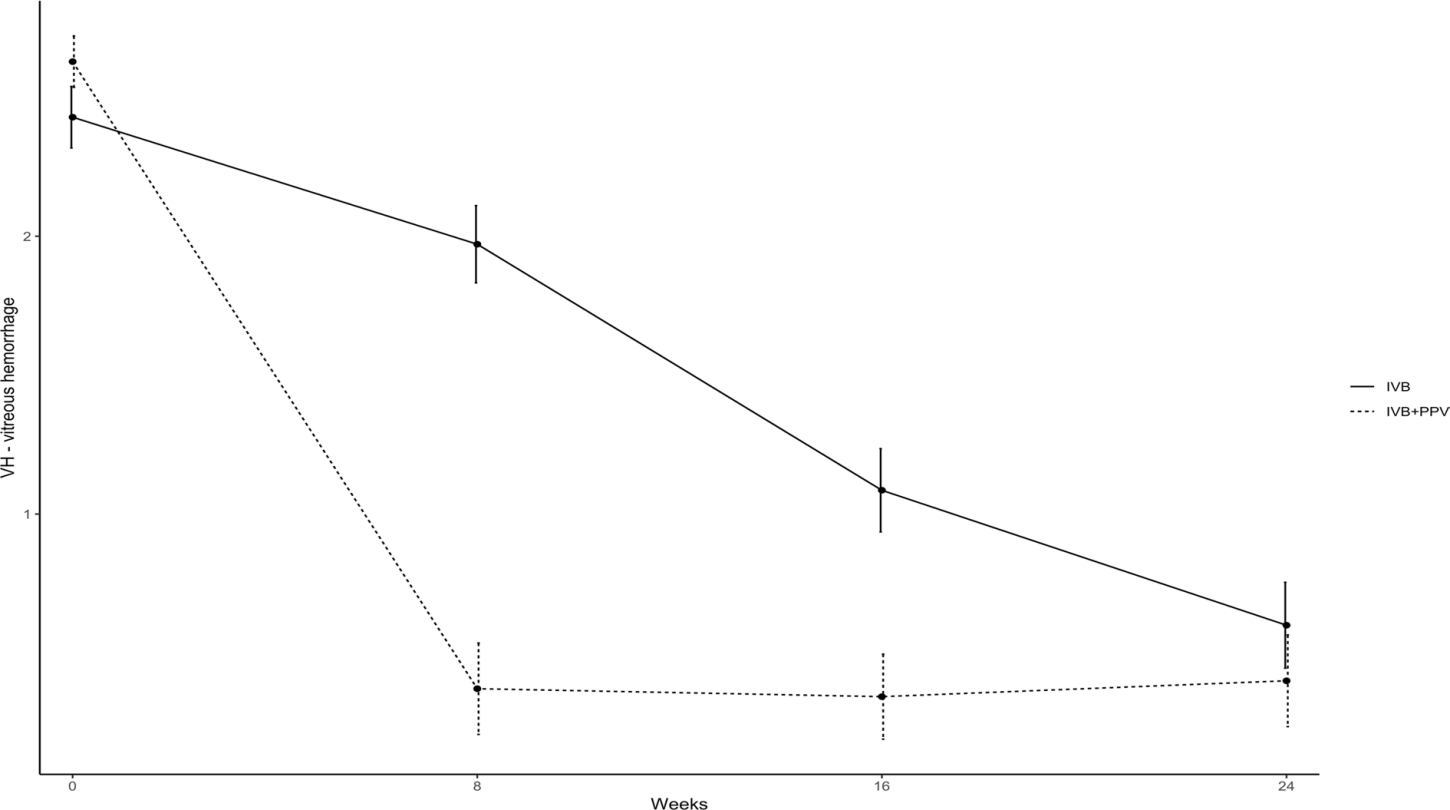
Vitreous hemorrhage score progression during the 24-week study period in both groups. Note the faster reduction in Group B score (these eyes were treated with IVB and PPV) when compared to Group A (these eyes were treated with IVB only). However, after 24 weeks, the mean score are similar in both groups

Mean VH score reduction (± SEM) of 0.4571 ± 0.0283 (*p* = 0.0014), 1.3429 ± 0.0393 (*p* < 0.0001) and 1.8286 ± 0.0438 (*p* < 0.001) was observed in Group A at 8, 16 and 24 weeks after treatment, respectively. (Table 2; Fig. 2) In Group B, the reduction in VH score (± SEM) was 2.2571 ± 0.0720 (*p* = 0.0014), 2.2857 ± 0.0606 (*p* < 0.0001) and 2.2286 ± 0.0726 (*p* < 0.001) at 8, 16 and 24 weeks after treatment, respectively. Group comparison revealed a significantly greater reduction in mean VH score in Group B at 8 and 16 weeks after treatment (*p* < 0.0001). However, at 24 weeks this difference was no longer statistically significant (*p* = 0.1854). (Table 3; Fig. 2).

**Table 2:** Progression of vitreous hemorrhage score during the study in Group A

Group A	Baseline	After 1st IVB (8 Weeks)	After 2nd IVB (16 Weeks)	After 3rd IVB (24 Weeks)
Grade 0	0	2	8	21
Grade 1	3	6	17	8
Grade 2	14	18	7	4
Grade 3	18	9	3	2

*IVB* intravitreous bevacizumab injection

**Table 3:** Progression of vitreous hemorrhage score during the study in Group B

Group B	Baseline	8 Weeks	16 Weeks	24 Weeks
Grade 0	0	31	31	30
Grade 1	1	1	1	1
Grade 2	11	0	0	0
Grade 3	23	3	3	4

### Best-corrected visual acuity (BCVA)

**Fig. 3:**
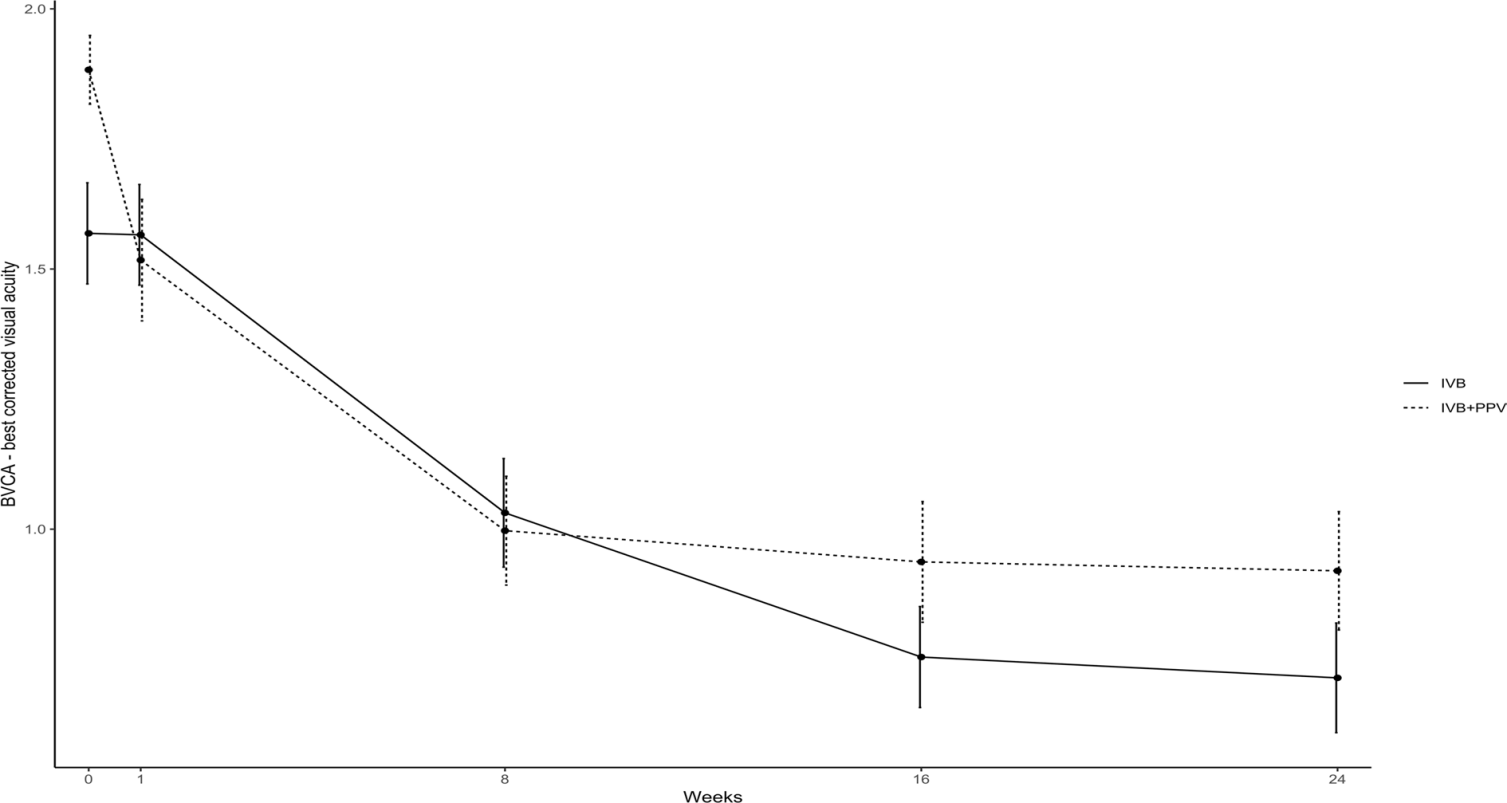
BCVA change progression during the 24-week study period in both groups. After one week of follow-up, there is significant improvement in BCVA only in the group which underwent pars plana vitrectomy (Group B). In the following visits until week 24, there was no difference in BCVA change between the groups

Mean logMAR BCVA (± SEM) was significantly higher at baseline in Group A (1.57 ± 0.10) when compared to Group B (1.88 ± 0.07) (*p* = 0.013). In Group A, mean (± SEM) logMAR BCVA showed an improvement of 0.00285 ± 0.0004 (*p* = 0.971), 0.5371 ± 0.0072 (*p* < 0.0001), 0.8143 ± 0.0001 (*p* < 0.0001) and 0.8543 ± 0.0008 (*p* < 0.0001) compared to baseline at 1, 8, 16 and 24 weeks after treatment, respectively. In Group B, mean (± SEM) logMAR BCVA showed an improvement of 0.3657 ± 0.0507 (*p* = 0.0002), 0.8857 ± 0.0385 (*p* < 0.0001), 0.9457 ± 0.0499 (*p* < 0.0001) and 0.9629 ± 0477 (*p* < 0.0001) compared to baseline at 1, 8, 16 and 24 weeks after treatment, respectively. No significant difference in logMAR BCVA improvement was observed between groups at 24 weeks after treatment. (Fig. 3).

### Intraocular pressure

We observed one case of increased intraocular pressure (IOP) > 21 mmHg in Group A; no IOP-lowering treatment was administered to any patients in this group. We also observed two cases of IOP > 21 mmHg in Group B; both of these patients were treated with timolol maleate and dorzolamide for 2–4 weeks, with a return to normal IOP levels.

### Recurrent VH

One eye in Group A developed recurrent VH at week 16. In contrast, 4 eyes in Group B developed recurrent VH prior to 8 weeks, and 1 additional eye developed recurrent VH at week 16. Due to failure of the recurrent VH to clear, repeat PPV was performed in 3 eyes after week 24.

### Adverse events

During the 24-week follow-up period, one eye in Group A developed TRD. None of the study eyes developed uveitis, endophthalmitis, ocular toxicity, or (for eyes in Group A) cataract progression.

## Discussion

To our knowledge, and based on a computerized search of the Medline database, the current study is the first to compare VH clearance in a prospective manner after treatment with IVB alone (Group A) versus IVB followed by PPV (Group B). The study demonstrated that the mean VH score was lower at 8 and 16 weeks in Group A, although the scores for the two groups were similar at 24 weeks. Thus, PPV was associated with more rapid clearance of the VH, but the final degree of VH clearance was similar in the two groups.

In Group A, the proportion of eyes with grade 3 VH decreased from 51.4% at baseline to 25.7%, 8.5% and 5.7% at 8, 16 and 24 weeks, respectively. At week 24, 60% of eyes in Group A had complete VH resolution, and 82.8% had grade 0 or grade 1 VH, which permitted application/completion of PRP. In Group B, the proportion of eyes with grade 3 VH decreased from 65.7% at baseline to 8.5, 8.5 and 11.4% after 8, 16 and 24 weeks, respectively. At week 24, 85.7% of eyes in Group B had complete VH resolution. Alagoz et al. [4] studied 2 groups of patients with VH, one of them treated with a single IVB bevacizumab and the other managed with observation. VH clearance time was defined as the time to when the vessels in the posterior pole and the optic disc were clearly visible and three or more peripheral retinal quadrants were sufficiently visible for the application of PRP, corresponding to grade 0 and 1 of VH in our study. The time for VH clearance in the group treated with IVB in the study by Alagoz et al. [4] was 9.2 ± 8.4 weeks, and this clearance occurred in 86.7% of the patients. Although the patients in the study by Alagoz et al. achieved VH resolution in a shorter period of time than did the patients in our study, the proportion with complete VH resolution was similar to that observed in our study; in our study, a VH grade of 0 or 1 was achieved in 71.4% of eyes by week 16 and 82.8% by week 24. In a study by Huang et al. [22] of patients with PDR-associated VH, patients were treated with one IVB and supplementary PRP when possible. A second IVB within 4–6 weeks (performed if no evidence of VH resolution was observed) was administered to 22.5% (9/40) of the patients. Patients in the second group (the control group) were observed. The time needed for VH clearance in the IVB group from Huang et al. was 12.6 ± 9.6 weeks (*p* = 0.02), with clearance occurring in 90% of cases. This was a higher clearance rate achieved at an earlier time than in our study, in which the VH clearance rate was 82.8% over a period of 24 weeks. This difference in VH clearance time and in the rate of VH clearance may be due to the lower percentage of patients with previous PRP in patients in our group A (45.7%) compared to 82% of patients with previous PRP in the IVB group from Huang et al.

The DRVS [14] included diabetic patients with VH of less than 6 months duration and reduced VA to less than 5/200 who were randomized to early PPV within 6 months or to PPV after 1 year. In the deferral group, in which patients were just observed, complete VH resolution without PPV or anti-VEGF injection occurred in 19.8% (61/308) of patients after 1 year of observation. Among the patients treated with early PPV, 77% achieved and maintained full VH clearance and the remaining 23% had recurrent VH compared to 14% in the group treated with PPV after 1 year. In our study, at 24 weeks, the rate of VH resolution was 82.8% in the group treated with PPV, with 8.5% (3/35) of the patients requiring reintervention after 6 months. Thus, there was a higher rate of VH clearance and a lower rate of reintervention in our study when compared to the DRVS, which may be explained by the current improvement in diabetes control, the development of the use of laser, and the evolution of the vitrectomy technique (preoperative use of antiangiogenic agents, instruments of lower caliber, and new machines that better control IOP and intraoperative bleeding).

The mean baseline BCVA differed between our study (group A 1.57 ± 0.10) and the IVB study groups published by Huang et al. (1.30 ± 0.5) and Alagoz et al. (1.83 ± 1.0). In our study, we observed an improvement of 0.72 ± 0.07 in Group A at 24 weeks compared to baseline. This difference was better than that reported by Huang et al., which was 0.45 ± 0.47 after a longer follow-up (48 weeks) compared to baseline. However, Alagoz et al. reported an improvement of VA of 1.05 ± 1.0 logMAR over a shorter follow-up period (14.5 ± 6.1 weeks), which was superior to that observed in other studies. It should be noted that the lower baseline BCVA in our study compared to that in the study by Huang et al. means there was more room for BCVA improvement among the patients in our study. The conversion of low BCVA scores to logMAR may also influence its baseline value. We based the counting fingers and hand motion visual acuity conversion on that suggested by Schulze-Bonsel et al. [23] and, although not identical, the conversion used by Huang et al. was similar. However, the conversion used by Alagoz et al. [4] was not mentioned, raising reservations when comparing the rates of improvement of that study to those of others. Finally, baseline BCVA may be influenced by other factors, such as macular edema and/or ischemia, and these factors were not controlled for in our study.

In Group B, at 24 weeks, visual acuity was better than 20/40 in 22.8% of the patients and better than 20/400 in 74.3%. In the DRVS [14], after the same period of 6 months, 24% of the patients had an acuity better than 20/40 and 50% had acuity better than 20/400 in the group treated with PPV. Thus, the proportion of patients with BCVA better than 20/400 was higher in the present study in comparison to the DRVS. The DRVS included only patients with vision worse than 5/200 (20/800), while in our study only 62.8% of patients had visual acuity within this range. This difference, as well as the differences inherent to the quality of the vitrectomy machines, instrumentation and techniques, complicates the comparison of these rates.

Many studies have shown NV regression with the use of an anti-VEGF agent. Jorge et al. (IBEPE study) [17] prospectively assessed the effects of a single IVB in patients with DR and NV refractory to PRP. One week after treatment, the active NV leakage demonstrated by fluorescein angiography was reduced in 11/15 (74%) patients and absent in the remaining 4 (26%). The absence of leakage was observed in all patients during the 6^th^ week, and a return of leakage was observed in 14/15 patients (93%) during the 12^th^ week after injection, although with a smaller mean area than that observed at baseline. A study by the same group [24] using ranibizumab yielded similar results. Thus, in the present study, an interval of 8 weeks between anti-VEGF injections was chosen because we felt this represents a safe interval between the 6 weeks reported in previous studies when NV disappeared completely and 12 weeks when return of NV was detected.

In Group B we administered IVB 7 days before PPV, consistent with information in the literature. Chen and Park [25] and Avery et al. [26] reported a reduction of intraoperative bleeding during PPV in patients with advanced PDR after IVB between 2 and 11 days before surgery. This is also a period of time compatible with the study by Ishikawa et al. [27], who suggested an interval of 7 days or less between IVB and PPV in order to reduce the risk of vitreoretinal traction. Lucena et al. [28] used an interval of 14 days between IVB and PPV for PDR with good results in reducing intraoperative bleeding.

In studies using an anti-VEGF agent for PDR and VH, it is important to select patients without obvious vitreoretinal adhesion and to monitor for possible development of TRD via serial clinical and echographic examinations [29]. Although we used echography to exclude retinal traction prior to intravitreal injection, we had 1 case of TRD, as was also observed by Huang et al. in the group treated with an anti-VEGF agent. No other adverse events related to intravitreal injection were observed in any patient during the 24 weeks of the study (no evidence of uveitis, endophthalmitis or ocular toxicity). Also, there were no significant lens changes in phakic patients, and no systemic adverse effects. Lastly, intraocular hypertension occurred in 1 and 2 patients from Groups A and B, respectively, and, in both groups, this may have been due to dehemoglobinized red blood cells (ghost cells) obstructing the trabecular meshwork [30].

In the present study, recurrent VH occurred in 1/35 (2.8%) Group A patients and in 5/35 (14.3%) Group B patients. We believe this difference between groups was due to the fact that Group B patients did not receive additional IVB over the 24 weeks of the study. The study of Alagoz et al. [4] reported recurrent VH in 4 patients of the group treated with IVB. The lower re-bleeding rate in the present study compared to Alagoz et al. [4] may be secondary to the fixed regimen of 3 anti-VEGF injections in our study Group A compared to a single application in the cited study.

The main parameter chosen for the present study was the change in VH score. This parameter seemed to be the most important objective of VH treatment that was not affected by other variables such as the presence of macular edema/ischemia or diabetic optic neuropathy. These variables would have great variability and influence parameters such as BCVA and macular thickness, and probably a very large sample of patients would be necessary to reach a significant and robust conclusion using these parameters. Despite the exploratory nature of our study, a significant difference between groups was detected for change in VH score during the study. The means and SEM may now be used to estimate the sample size of future studies.

The VH score improved in both groups. In the group treated with three applications of IVB at 8-week intervals, 82.8% of eyes had sufficient clearing of VH to permit the application of PRP and 60% demonstrated complete VH resolution within 24 weeks, while 85.7% of eyes in the IVB plus PPV group achieved complete VH clearance. Among the advantages of treatment with IVB alone is the preservation of the vitreous, which would be important for future intravitreal anti-VEGF injections for persistent NV or associated macular edema. A benefit of PPV is more rapid VH clearance occurring at higher rates, but with the associated risks of PPV.

## Conclusion

PPV with preoperative IVB is associated with more rapid clearance of VH and improvement in BCVA than IVB injections alone. However, after 24 weeks of follow-up, the reduction in VH score and BCVA were similar between both treatment strategies.

## Data Availability

The datasets used and/or analyzed during the current study are available from the corresponding author on reasonable request.

## Abbreviations

OD: Right eye
OS: Left eye
SD-OCT: Spectral domain optical coherence tomography
DR: Diabetic retinopathy
DM: Diabetes mellitus
NV: Neovascularization
PDR: Proliferative diabetic retinopathy
PRP: Panretinal laser photocoagulation
VEGF: Vascular endothelial growth factor
VH: Vitreous hemorrhage
DRVS: Diabetic Retinopathy Vitrectomy Study
PPV: Pars plana vitrectomy
IVB: Intravitreal injections of bevacizumab
BCVA: Best-corrected visual acuity
ETDRS: Early Treatment Diabetic Retinopathy Study
BIO: Binocular indirect ophthalmoscope
